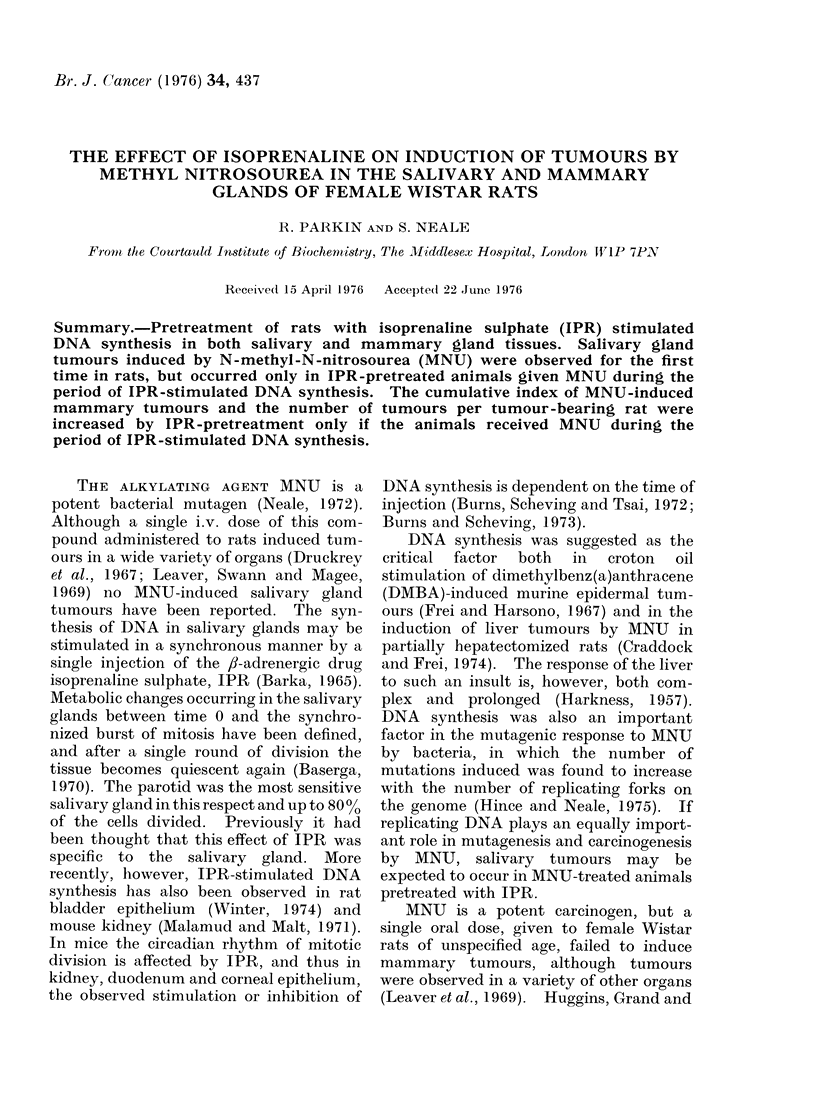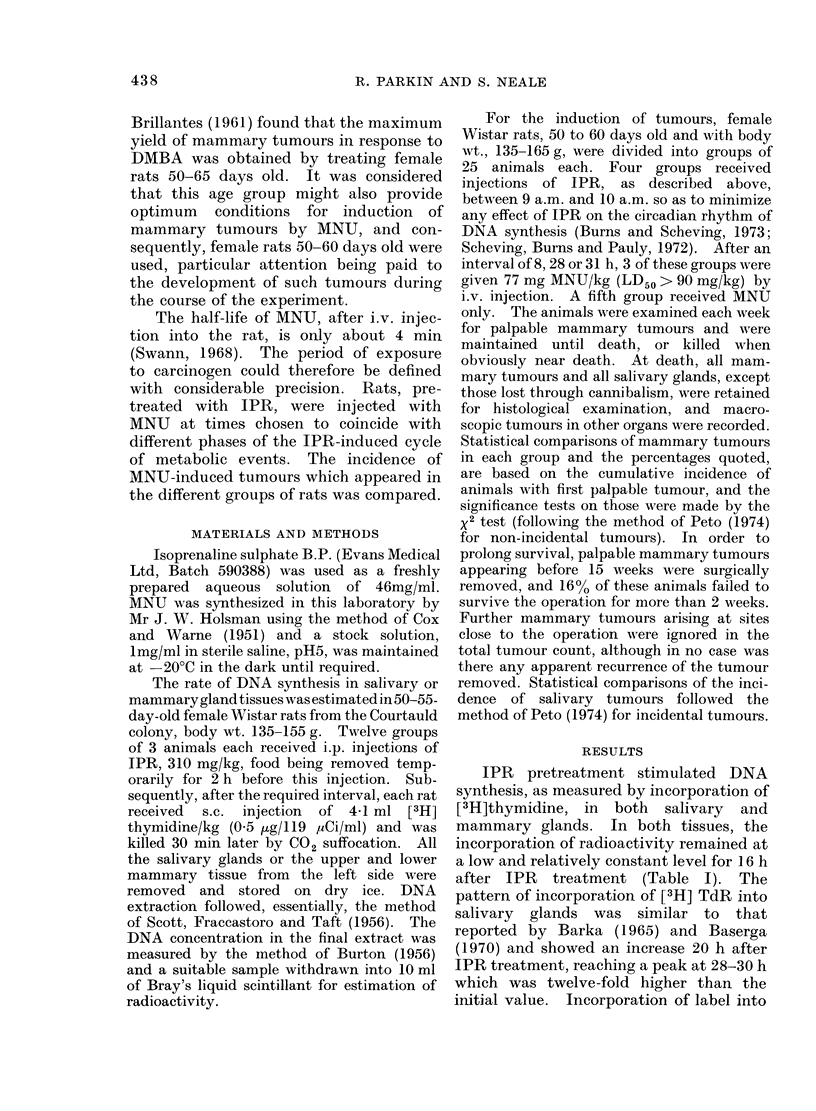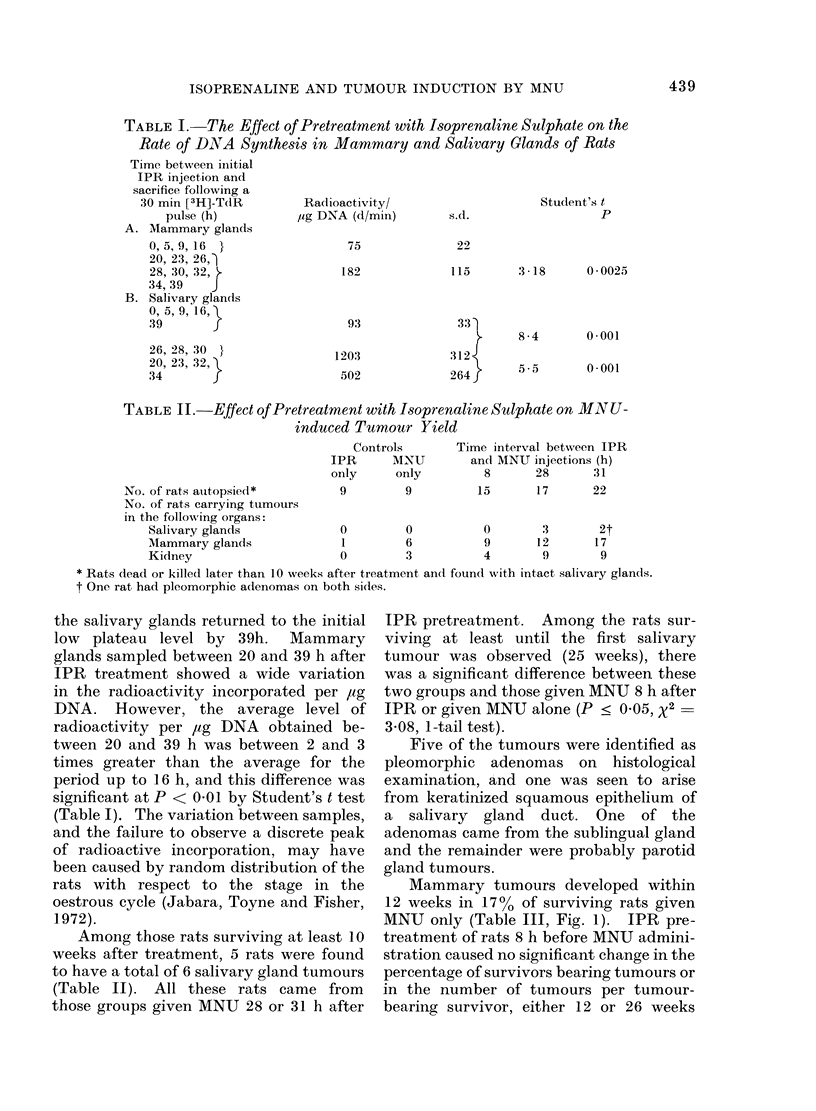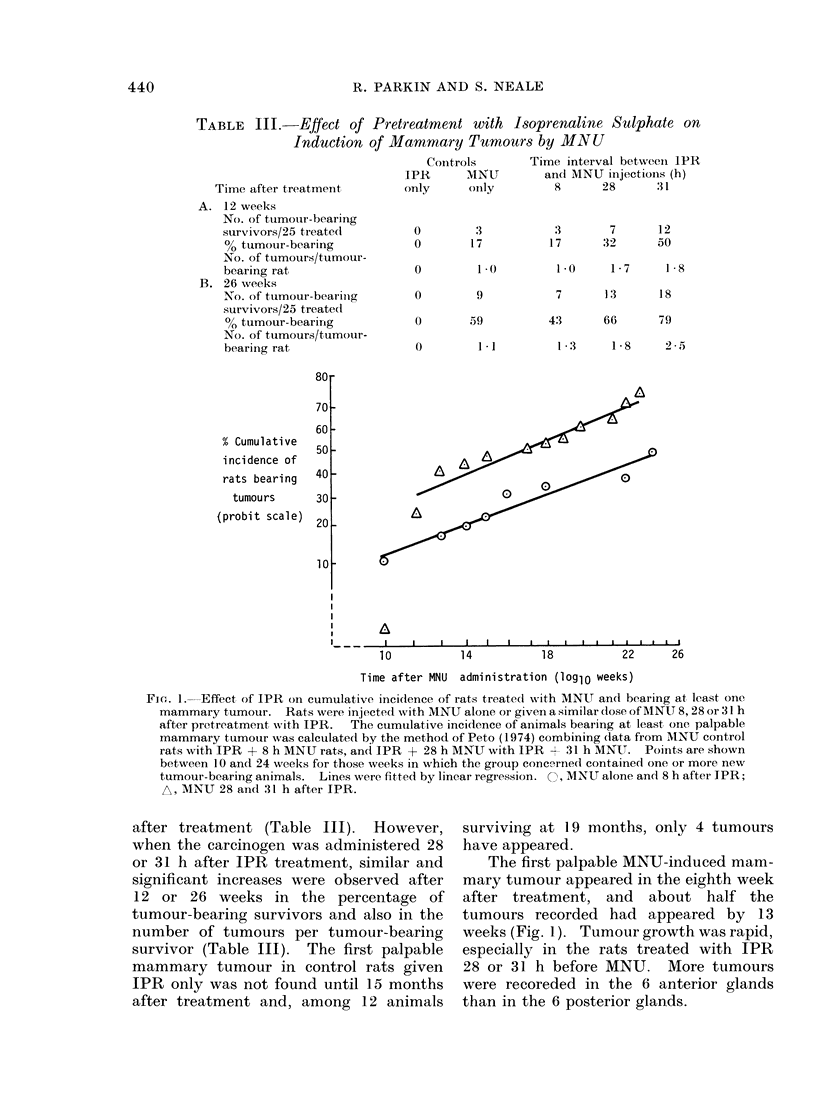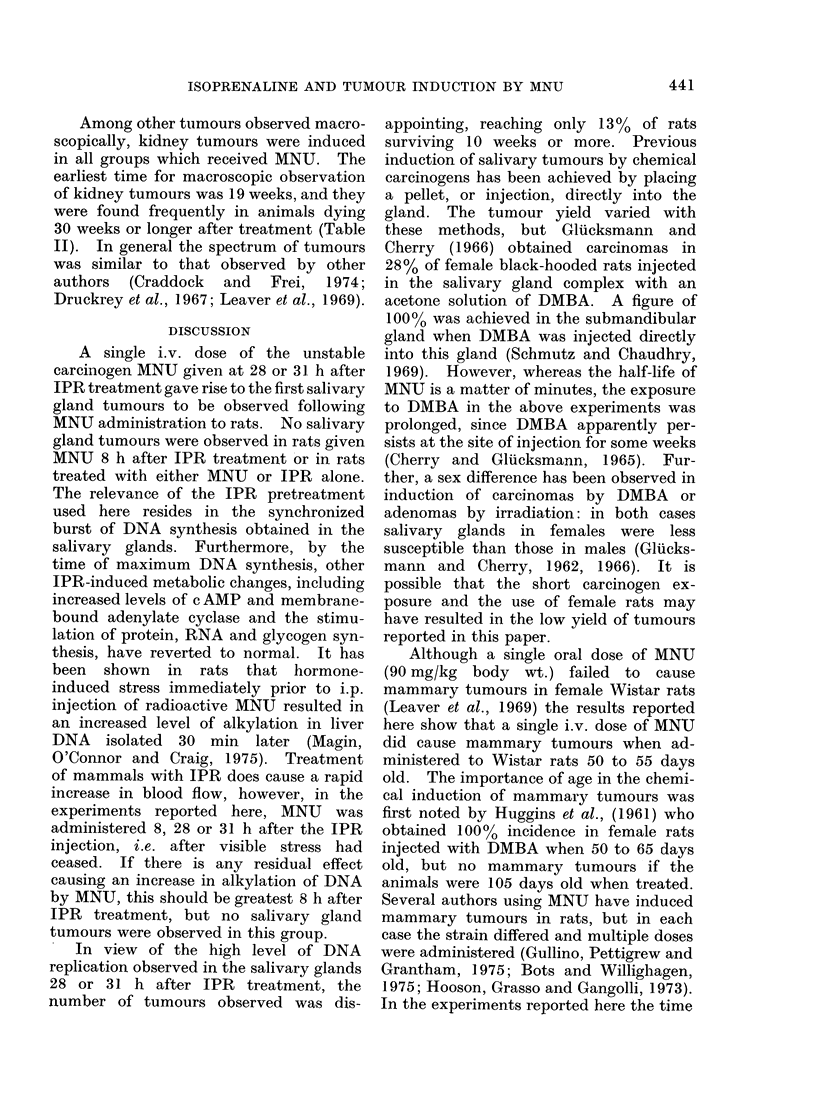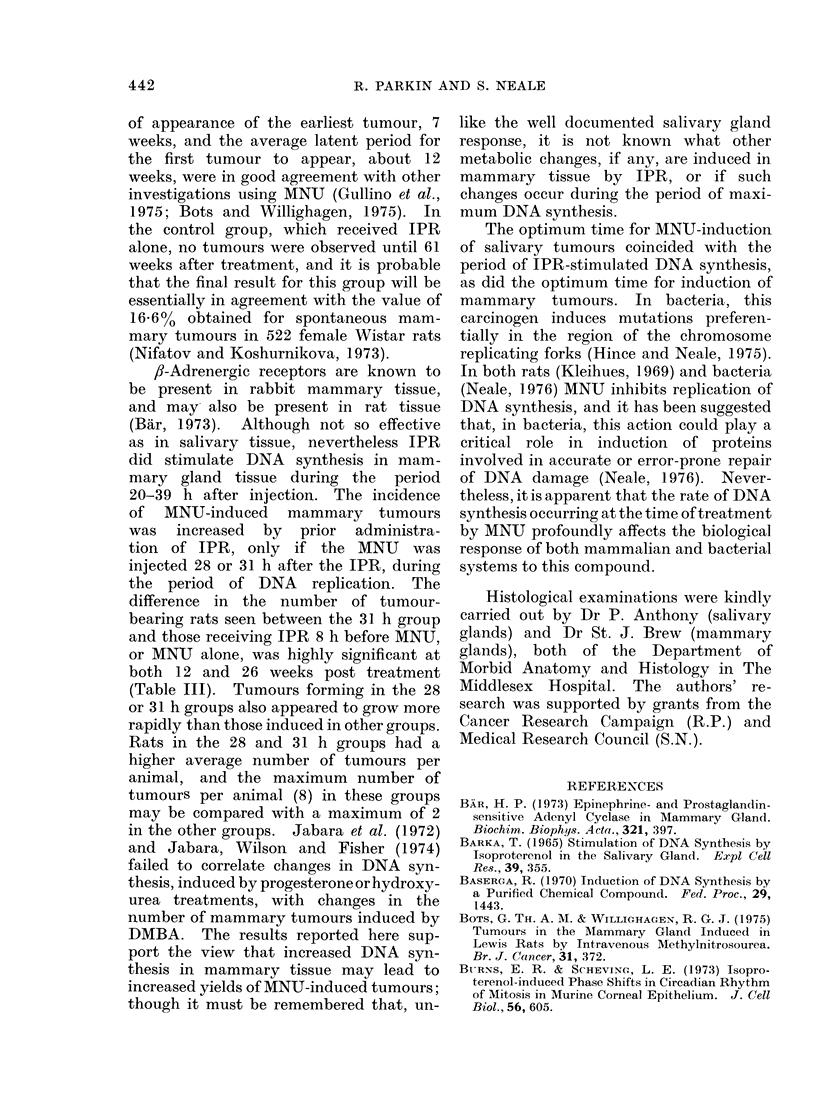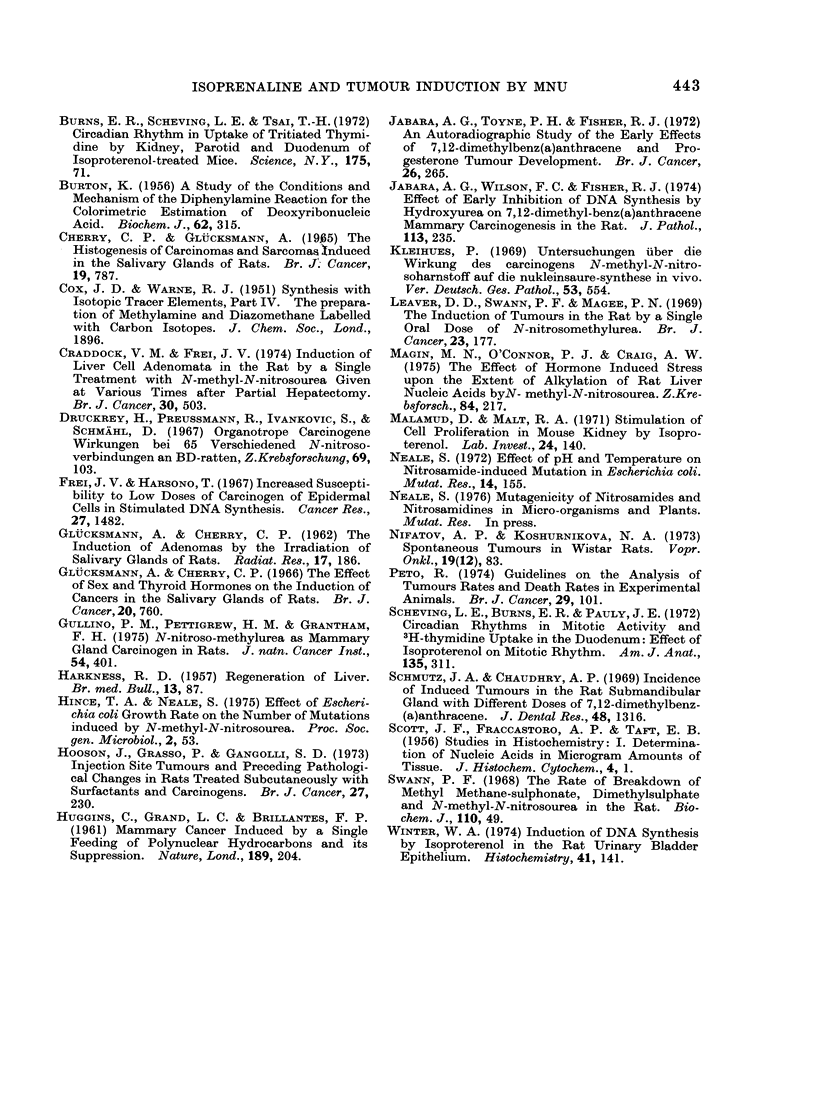# The effect of isoprenaline on induction of tumours by methyl nitrosourea in the salivary and mammary glands of female wistar rats.

**DOI:** 10.1038/bjc.1976.189

**Published:** 1976-10

**Authors:** R. Parkin, S. Neale

## Abstract

Pretreatment of rats with isoprenaline sulphate (IPR) stimulated DNA synthesis in both salivary and mammary gland tissues. Salivary gland tumours induced by N-methyl-N-nitrosourea (MNU) were observed for the first time in rats, but occurred only in IPR-pretreated animals given MNU during the period of IPR-stimulated DNA synthesis. The cumulative index of MNU-induced mammary tumours and the number of tumours per tumour-bearing rat were increased by IPR-pretreament only if the animals received MNU during the period of IPR-stimulated DNA synthesis.


*Br. J. Cancer* (1976) **34,** 437

### THE EFFECT OF ISOPRENALINE ON INDUCTION OF TUMOURS BY METHYL NITROSOUREA IN THE SALIVARY AND MAMMARY GLANDS OF FEMALE WISTAR RATS

R. PARKIN AND S. NEALE

*From the Courtauld Institute of Biochemistry, The Middlesex Hospital, London W1P 7PN*

Received 15 April 1976 Accepted 22 June 1976

**Summary.—Pretreatment of rats with isoprenaline sulphate (IPR) stimulated DNA synthesis in both salivary and mammary gland tissues. Salivary gland tumours induced by N-methyl-N-nitrosourea (MNU) were observed for the first time in rats, but occurred only in IPR-pretreated animals given MNU during the period of IPR-stimulated DNA synthesis. The cumulative index of MNU-induced mammary tumours and the number of tumours per tumour-bearing rat were increased by IPR-pretreatment only if the animals received MNU during the period of IPR-stimulated DNA synthesis.**

THE ALKYLATING AGENT MNU is a potent bacterial mutagen (Neale, 1972). Although a single i.v. dose of this compound administered to rats induced tumours in a wide variety of organs (Druckrey *et al.*, 1967; Leaver, Swann and Magee, 1969) no MNU-induced salivary gland tumours have been reported. The synthesis of DNA in salivary glands may be stimulated in a synchronous manner by a single injection of the $\beta$-adrenergic drug isoprenaline sulphate, IPR (Barka, 1965). Metabolic changes occurring in the salivary glands between time 0 and the synchronized burst of mitosis have been defined, and after a single round of division the tissue becomes quiescent again (Baserga, 1970). The parotid was the most sensitive salivary gland in this respect and up to 80% of the cells divided. Previously it had been thought that this effect of IPR was specific to the salivary gland. More recently, however, IPR-stimulated DNA synthesis has also been observed in rat bladder epithelium (Winter, 1974) and mouse kidney (Malamud and Malt, 1971). In mice the circadian rhythm of mitotic division is affected by IPR, and thus in kidney, duodenum and corneal epithelium, the observed stimulation or inhibition of DNA synthesis is dependent on the time of injection (Burns, Scheving and Tsai, 1972; Burns and Scheving, 1973).

DNA synthesis was suggested as the critical factor both in croton oil stimulation of dimethylbenz(a)anthracene (DMBA)-induced murine epidermal tumours (Frei and Harsono, 1967) and in the induction of liver tumours by MNU in partially hepatectomized rats (Craddock and Frei, 1974). The response of the liver to such an insult is, however, both complex and prolonged (Harkness, 1957). DNA synthesis was also an important factor in the mutagenic response to MNU by bacteria, in which the number of mutations induced was found to increase with the number of replicating forks on the genome (Hince and Neale, 1975). If replicating DNA plays an equally important role in mutagenesis and carcinogenesis by MNU, salivary tumours may be expected to occur in MNU-treated animals pretreated with IPR.

MNU is a potent carcinogen, but a single oral dose, given to female Wistar rats of unspecified age, failed to induce mammary tumours, although tumours were observed in a variety of other organs (Leaver *et al.*, 1969). Huggins, Grand and

Brillantes (1961) found that the maximum yield of mammary tumours in response to DMBA was obtained by treating female rats 50–65 days old. It was considered that this age group might also provide optimum conditions for induction of mammary tumours by MNU, and consequently, female rats 50–60 days old were used, particular attention being paid to the development of such tumours during the course of the experiment.

The half-life of MNU, after i.v. injection into the rat, is only about 4 min (Swann, 1968). The period of exposure to carcinogen could therefore be defined with considerable precision. Rats, pretreated with IPR, were injected with MNU at times chosen to coincide with different phases of the IPR-induced cycle of metabolic events. The incidence of MNU-induced tumours which appeared in the different groups of rats was compared.

### MATERIALS AND METHODS

Isoprenaline sulphate B.P. (Evans Medical Ltd, Batch 590388) was used as a freshly prepared aqueous solution of 46mg/ml. MNU was synthesized in this laboratory by Mr J. W. Holsman using the method of Cox and Warne (1951) and a stock solution, 1mg/ml in sterile saline, pH5, was maintained at −20°C in the dark until required.

The rate of DNA synthesis in salivary or mammary gland tissues was estimated in 50–55-day-old female Wistar rats from the Courtauld colony, body wt. 135–155 g. Twelve groups of 3 animals each received i.p. injections of IPR, 310 mg/kg, food being removed temporarily for 2 h before this injection. Subsequently, after the required interval, each rat received s.c. injection of 4·1 ml [$^3$H] thymidine/kg (0·5 $\mu$g/119 $\mu$Ci/ml) and was killed 30 min later by $CO_2$ suffocation. All the salivary glands or the upper and lower mammary tissue from the left side were removed and stored on dry ice. DNA extraction followed, essentially, the method of Scott, Fraccastoro and Taft (1956). The DNA concentration in the final extract was measured by the method of Burton (1956) and a suitable sample withdrawn into 10 ml of Bray's liquid scintillant for estimation of radioactivity.

For the induction of tumours, female Wistar rats, 50 to 60 days old and with body wt., 135–165 g, were divided into groups of 25 animals each. Four groups received injections of IPR, as described above, between 9 a.m. and 10 a.m. so as to minimize any effect of IPR on the circadian rhythm of DNA synthesis (Burns and Scheving, 1973; Scheving, Burns and Pauly, 1972). After an interval of 8, 28 or 31 h, 3 of these groups were given 77 mg MNU/kg ($LD_{50} > 90$ mg/kg) by i.v. injection. A fifth group received MNU only. The animals were examined each week for palpable mammary tumours and were maintained until death, or killed when obviously near death. At death, all mammary tumours and all salivary glands, except those lost through cannibalism, were retained for histological examination, and macroscopic tumours in other organs were recorded. Statistical comparisons of mammary tumours in each group and the percentages quoted, are based on the cumulative incidence of animals with first palpable tumour, and the significance tests on those were made by the $\chi^2$ test (following the method of Peto (1974) for non-incidental tumours). In order to prolong survival, palpable mammary tumours appearing before 15 weeks were surgically removed, and 16% of these animals failed to survive the operation for more than 2 weeks. Further mammary tumours arising at sites close to the operation were ignored in the total tumour count, although in no case was there any apparent recurrence of the tumour removed. Statistical comparisons of the incidence of salivary tumours followed the method of Peto (1974) for incidental tumours.

### RESULTS

IPR pretreatment stimulated DNA synthesis, as measured by incorporation of [$^3$H]thymidine, in both salivary and mammary glands. In both tissues, the incorporation of radioactivity remained at a low and relatively constant level for 16 h after IPR treatment (Table I). The pattern of incorporation of [$^3$H] TdR into salivary glands was similar to that reported by Barka (1965) and Baserga (1970) and showed an increase 20 h after IPR treatment, reaching a peak at 28–30 h which was twelve-fold higher than the initial value. Incorporation of label into

TABLE I.—*The Effect of Pretreatment with Isoprenaline Sulphate on the Rate of DNA Synthesis in Mammary and Salivary Glands of Rats*

| Time between initial IPR injection and sacrifice following a 30 min [$^3$H]-TdR pulse (h) | Radioactivity/ $\mu$g DNA (d/min) | s.d. | Student's *t* | *P* |
|---|---|---|---|---|
| A. Mammary glands | | | | |
| 0, 5, 9, 16 | 75 | 22 | | |
| 20, 23, 26, 28, 30, 32, 34, 39 | 182 | 115 | 3·18 | 0·0025 |
| B. Salivary glands | | | | |
| 0, 5, 9, 16, 39 | 93 | 33 | | |
| 26, 28, 30 | 1203 | 312 | 8·4 | 0·001 |
| 20, 23, 32, 34 | 502 | 264 | 5·5 | 0·001 |

TABLE II.—*Effect of Pretreatment with Isoprenaline Sulphate on MNU-induced Tumour Yield*

| | Controls | | Time interval between IPR and MNU injections (h) | | |
|---|---|---|---|---|---|
| | IPR only | MNU only | 8 | 28 | 31 |
| No. of rats autopsied* | 9 | 9 | 15 | 17 | 22 |
| No. of rats carrying tumours in the following organs: | | | | | |
| Salivary glands | 0 | 0 | 0 | 3 | 2† |
| Mammary glands | 1 | 6 | 9 | 12 | 17 |
| Kidney | 0 | 3 | 4 | 9 | 9 |

* Rats dead or killed later than 10 weeks after treatment and found with intact salivary glands.
† One rat had pleomorphic adenomas on both sides.

the salivary glands returned to the initial low plateau level by 39h. Mammary glands sampled between 20 and 39 h after IPR treatment showed a wide variation in the radioactivity incorporated per $\mu$g DNA. However, the average level of radioactivity per $\mu$g DNA obtained between 20 and 39 h was between 2 and 3 times greater than the average for the period up to 16 h, and this difference was significant at $P < 0{\cdot}01$ by Student's *t* test (Table I). The variation between samples, and the failure to observe a discrete peak of radioactive incorporation, may have been caused by random distribution of the rats with respect to the stage in the oestrous cycle (Jabara, Toyne and Fisher, 1972).

Among those rats surviving at least 10 weeks after treatment, 5 rats were found to have a total of 6 salivary gland tumours (Table II). All these rats came from those groups given MNU 28 or 31 h after IPR pretreatment. Among the rats surviving at least until the first salivary tumour was observed (25 weeks), there was a significant difference between these two groups and those given MNU 8 h after IPR or given MNU alone ($P \leq 0{\cdot}05$, $\chi^2 = 3{\cdot}08$, 1-tail test).

Five of the tumours were identified as pleomorphic adenomas on histological examination, and one was seen to arise from keratinized squamous epithelium of a salivary gland duct. One of the adenomas came from the sublingual gland and the remainder were probably parotid gland tumours.

Mammary tumours developed within 12 weeks in 17% of surviving rats given MNU only (Table III, Fig. 1). IPR pretreatment of rats 8 h before MNU administration caused no significant change in the percentage of survivors bearing tumours or in the number of tumours per tumour-bearing survivor, either 12 or 26 weeks

TABLE III.—*Effect of Pretreatment with Isoprenaline Sulphate on Induction of Mammary Tumours by MNU*

| Time after treatment | Controls IPR only | MNU only | Time interval between IPR and MNU injections (h) 8 | 28 | 31 |
|---|---|---|---|---|---|
| A. 12 weeks | | | | | |
| No. of tumour-bearing survivors/25 treated | 0 | 3 | 3 | 7 | 12 |
| % tumour-bearing | 0 | 17 | 17 | 32 | 50 |
| No. of tumours/tumour-bearing rat | 0 | 1·0 | 1·0 | 1·7 | 1·8 |
| B. 26 weeks | | | | | |
| No. of tumour-bearing survivors/25 treated | 0 | 9 | 7 | 13 | 18 |
| % tumour-bearing | 0 | 59 | 43 | 66 | 79 |
| No. of tumours/tumour-bearing rat | 0 | 1·1 | 1·3 | 1·8 | 2·5 |

FIG. 1.—Effect of IPR on cumulative incidence of rats treated with MNU and bearing at least one mammary tumour. Rats were injected with MNU alone or given a similar dose of MNU 8, 28 or 31 h after pretreatment with IPR. The cumulative incidence of animals bearing at least one palpable mammary tumour was calculated by the method of Peto (1974) combining data from MNU control rats with IPR + 8 h MNU rats, and IPR + 28 h MNU with IPR + 31 h MNU. Points are shown between 10 and 24 weeks for those weeks in which the group concerned contained one or more new tumour-bearing animals. Lines were fitted by linear regression. ○, MNU alone and 8 h after IPR; △, MNU 28 and 31 h after IPR.

after treatment (Table III). However, when the carcinogen was administered 28 or 31 h after IPR treatment, similar and significant increases were observed after 12 or 26 weeks in the percentage of tumour-bearing survivors and also in the number of tumours per tumour-bearing survivor (Table III). The first palpable mammary tumour in control rats given IPR only was not found until 15 months after treatment and, among 12 animals surviving at 19 months, only 4 tumours have appeared.

The first palpable MNU-induced mammary tumour appeared in the eighth week after treatment, and about half the tumours recorded had appeared by 13 weeks (Fig. 1). Tumour growth was rapid, especially in the rats treated with IPR 28 or 31 h before MNU. More tumours were recoreded in the 6 anterior glands than in the 6 posterior glands.

Among other tumours observed macroscopically, kidney tumours were induced in all groups which received MNU. The earliest time for macroscopic observation of kidney tumours was 19 weeks, and they were found frequently in animals dying 30 weeks or longer after treatment (Table II). In general the spectrum of tumours was similar to that observed by other authors (Craddock and Frei, 1974; Druckrey *et al.*, 1967; Leaver *et al.*, 1969).

### DISCUSSION

A single i.v. dose of the unstable carcinogen MNU given at 28 or 31 h after IPR treatment gave rise to the first salivary gland tumours to be observed following MNU administration to rats. No salivary gland tumours were observed in rats given MNU 8 h after IPR treatment or in rats treated with either MNU or IPR alone. The relevance of the IPR pretreatment used here resides in the synchronized burst of DNA synthesis obtained in the salivary glands. Furthermore, by the time of maximum DNA synthesis, other IPR-induced metabolic changes, including increased levels of c AMP and membrane-bound adenylate cyclase and the stimulation of protein, RNA and glycogen synthesis, have reverted to normal. It has been shown in rats that hormone-induced stress immediately prior to i.p. injection of radioactive MNU resulted in an increased level of alkylation in liver DNA isolated 30 min later (Magin, O'Connor and Craig, 1975). Treatment of mammals with IPR does cause a rapid increase in blood flow, however, in the experiments reported here, MNU was administered 8, 28 or 31 h after the IPR injection, *i.e.* after visible stress had ceased. If there is any residual effect causing an increase in alkylation of DNA by MNU, this should be greatest 8 h after IPR treatment, but no salivary gland tumours were observed in this group.

In view of the high level of DNA replication observed in the salivary glands 28 or 31 h after IPR treatment, the number of tumours observed was disappointing, reaching only 13% of rats surviving 10 weeks or more. Previous induction of salivary tumours by chemical carcinogens has been achieved by placing a pellet, or injection, directly into the gland. The tumour yield varied with these methods, but Glücksmann and Cherry (1966) obtained carcinomas in 28% of female black-hooded rats injected in the salivary gland complex with an acetone solution of DMBA. A figure of 100% was achieved in the submandibular gland when DMBA was injected directly into this gland (Schmutz and Chaudhry, 1969). However, whereas the half-life of MNU is a matter of minutes, the exposure to DMBA in the above experiments was prolonged, since DMBA apparently persists at the site of injection for some weeks (Cherry and Glücksmann, 1965). Further, a sex difference has been observed in induction of carcinomas by DMBA or adenomas by irradiation: in both cases salivary glands in females were less susceptible than those in males (Glücksmann and Cherry, 1962, 1966). It is possible that the short carcinogen exposure and the use of female rats may have resulted in the low yield of tumours reported in this paper.

Although a single oral dose of MNU (90 mg/kg body wt.) failed to cause mammary tumours in female Wistar rats (Leaver *et al.*, 1969) the results reported here show that a single i.v. dose of MNU did cause mammary tumours when administered to Wistar rats 50 to 55 days old. The importance of age in the chemical induction of mammary tumours was first noted by Huggins *et al.*, (1961) who obtained 100% incidence in female rats injected with DMBA when 50 to 65 days old, but no mammary tumours if the animals were 105 days old when treated. Several authors using MNU have induced mammary tumours in rats, but in each case the strain differed and multiple doses were administered (Gullino, Pettigrew and Grantham, 1975; Bots and Willighagen, 1975; Hooson, Grasso and Gangolli, 1973). In the experiments reported here the time of appearance of the earliest tumour, 7 weeks, and the average latent period for the first tumour to appear, about 12 weeks, were in good agreement with other investigations using MNU (Gullino *et al.*, 1975; Bots and Willighagen, 1975). In the control group, which received IPR alone, no tumours were observed until 61 weeks after treatment, and it is probable that the final result for this group will be essentially in agreement with the value of 16·6% obtained for spontaneous mammary tumours in 522 female Wistar rats (Nifatov and Koshurnikova, 1973).

$\beta$-Adrenergic receptors are known to be present in rabbit mammary tissue, and may also be present in rat tissue (Bär, 1973). Although not so effective as in salivary tissue, nevertheless IPR did stimulate DNA synthesis in mammary gland tissue during the period 20–39 h after injection. The incidence of MNU-induced mammary tumours was increased by prior administration of IPR, only if the MNU was injected 28 or 31 h after the IPR, during the period of DNA replication. The difference in the number of tumour-bearing rats seen between the 31 h group and those receiving IPR 8 h before MNU, or MNU alone, was highly significant at both 12 and 26 weeks post treatment (Table III). Tumours forming in the 28 or 31 h groups also appeared to grow more rapidly than those induced in other groups. Rats in the 28 and 31 h groups had a higher average number of tumours per animal, and the maximum number of tumours per animal (8) in these groups may be compared with a maximum of 2 in the other groups. Jabara *et al.* (1972) and Jabara, Wilson and Fisher (1974) failed to correlate changes in DNA synthesis, induced by progesterone or hydroxyurea treatments, with changes in the number of mammary tumours induced by DMBA. The results reported here support the view that increased DNA synthesis in mammary tissue may lead to increased yields of MNU-induced tumours; though it must be remembered that, unlike the well documented salivary gland response, it is not known what other metabolic changes, if any, are induced in mammary tissue by IPR, or if such changes occur during the period of maximum DNA synthesis.

The optimum time for MNU-induction of salivary tumours coincided with the period of IPR-stimulated DNA synthesis, as did the optimum time for induction of mammary tumours. In bacteria, this carcinogen induces mutations preferentially in the region of the chromosome replicating forks (Hince and Neale, 1975). In both rats (Kleihues, 1969) and bacteria (Neale, 1976) MNU inhibits replication of DNA synthesis, and it has been suggested that, in bacteria, this action could play a critical role in induction of proteins involved in accurate or error-prone repair of DNA damage (Neale, 1976). Nevertheless, it is apparent that the rate of DNA synthesis occurring at the time of treatment by MNU profoundly affects the biological response of both mammalian and bacterial systems to this compound.

Histological examinations were kindly carried out by Dr P. Anthony (salivary glands) and Dr St. J. Brew (mammary glands), both of the Department of Morbid Anatomy and Histology in The Middlesex Hospital. The authors' research was supported by grants from the Cancer Research Campaign (R.P.) and Medical Research Council (S.N.).

### REFERENCES

Bär, H. P. (1973) Epinephrine- and Prostaglandin-sensitive Adenyl Cyclase in Mammary Gland. *Biochim. Biophys. Acta.*, **321**, 397.

Barka, T. (1965) Stimulation of DNA Synthesis by Isoproterenol in the Salivary Gland. *Expl Cell Res.*, **39**, 355.

Baserga, R. (1970) Induction of DNA Synthesis by a Purified Chemical Compound. *Fed. Proc.*, **29**, 1443.

Bots, G. Th. A. M. & Willighagen, R. G. J. (1975) Tumours in the Mammary Gland Induced in Lewis Rats by Intravenous Methylnitrosourea. *Br. J. Cancer*, **31**, 372.

Burns, E. R. & Scheving, L. E. (1973) Isoproterenol-induced Phase Shifts in Circadian Rhythm of Mitosis in Murine Corneal Epithelium. *J. Cell Biol.*, **56**, 605.

BURNS, E. R., SCHEVING, L. E. & TSAI, T.-H. (1972) Circadian Rhythm in Uptake of Tritiated Thymidine by Kidney, Parotid and Duodenum of Isoproterenol-treated Mice. *Science, N.Y.*, **175**, 71.

BURTON, K. (1956) A Study of the Conditions and Mechanism of the Diphenylamine Reaction for the Colorimetric Estimation of Deoxyribonucleic Acid. *Biochem. J.*, **62**, 315.

CHERRY, C. P. & GLÜCKSMANN, A. (1965) The Histogenesis of Carcinomas and Sarcomas Induced in the Salivary Glands of Rats. *Br. J. Cancer*, **19**, 787.

COX, J. D. & WARNE, R. J. (1951) Synthesis with Isotopic Tracer Elements, Part IV. The preparation of Methylamine and Diazomethane Labelled with Carbon Isotopes. *J. Chem. Soc., Lond.*, 1896.

CRADDOCK, V. M. & FREI, J. V. (1974) Induction of Liver Cell Adenomata in the Rat by a Single Treatment with *N*-methyl-*N*-nitrosourea Given at Various Times after Partial Hepatectomy. *Br. J. Cancer*, **30**, 503.

DRUCKREY, H., PREUSSMANN, R., IVANKOVIC, S., & SCHMÄHL, D. (1967) Organotrope Carcinogene Wirkungen bei 65 Verschiedened *N*-nitroso-verbindungen an BD-ratten, *Z.Krebsforschung*, **69**, 103.

FREI, J. V. & HARSONO, T. (1967) Increased Susceptibility to Low Doses of Carcinogen of Epidermal Cells in Stimulated DNA Synthesis. *Cancer Res.*, **27**, 1482.

GLÜCKSMANN, A. & CHERRY, C. P. (1962) The Induction of Adenomas by the Irradiation of Salivary Glands of Rats. *Radiat. Res.*, **17**, 186.

GLÜCKSMANN, A. & CHERRY, C. P. (1966) The Effect of Sex and Thyroid Hormones on the Induction of Cancers in the Salivary Glands of Rats. *Br. J. Cancer*, **20**, 760.

GULLINO, P. M., PETTIGREW, H. M. & GRANTHAM, F. H. (1975) *N*-nitroso-methylurea as Mammary Gland Carcinogen in Rats. *J. natn. Cancer Inst.*, **54**, 401.

HARKNESS, R. D. (1957) Regeneration of Liver. *Br. med. Bull.*, **13**, 87.

HINCE, T. A. & NEALE, S. (1975) Effect of *Escherichia coli* Growth Rate on the Number of Mutations induced by *N*-methyl-*N*-nitrosourea. *Proc. Soc. gen. Microbiol.*, **2**, 53.

HOOSON, J., GRASSO, P. & GANGOLLI, S. D. (1973) Injection Site Tumours and Preceding Pathological Changes in Rats Treated Subcutaneously with Surfactants and Carcinogens. *Br. J. Cancer*, **27**, 230.

HUGGINS, C., GRAND, L. C. & BRILLANTES, F. P. (1961) Mammary Cancer Induced by a Single Feeding of Polynuclear Hydrocarbons and its Suppression. *Nature, Lond.*, **189**, 204.

JABARA, A. G., TOYNE, P. H. & FISHER, R. J. (1972) An Autoradiographic Study of the Early Effects of 7,12-dimethylbenz(a)anthracene and Progesterone Tumour Development. *Br. J. Cancer*, **26**, 265.

JABARA, A. G., WILSON, F. C. & FISHER, R. J. (1974) Effect of Early Inhibition of DNA Synthesis by Hydroxyurea on 7,12-dimethyl-benz(a)anthracene Mammary Carcinogenesis in the Rat. *J. Pathol.*, **113**, 235.

KLEIHUES, P. (1969) Untersuchungen über die Wirkung des carcinogens *N*-methyl-*N*-nitrosoharnstoff auf die nukleinsaure-synthese in vivo. *Ver. Deutsch. Ges. Pathol.*, **53**, 554.

LEAVER, D. D., SWANN, P. F. & MAGEE, P. N. (1969) The Induction of Tumours in the Rat by a Single Oral Dose of *N*-nitrosomethylurea. *Br. J. Cancer*, **23**, 177.

MAGIN, M. N., O'CONNOR, P. J. & CRAIG, A. W. (1975) The Effect of Hormone Induced Stress upon the Extent of Alkylation of Rat Liver Nucleic Acids by*N*- methyl-*N*-nitrosourea. *Z.Krebsforsch.*, **84**, 217.

MALAMUD, D. & MALT, R. A. (1971) Stimulation of Cell Proliferation in Mouse Kidney by Isoproterenol. *Lab. Invest.*, **24**, 140.

NEALE, S. (1972) Effect of pH and Temperature on Nitrosamide-induced Mutation in *Escherichia coli. Mutat. Res.*, **14**, 155.

NEALE, S. (1976) Mutagenicity of Nitrosamides and Nitrosamidines in Micro-organisms and Plants. *Mutat. Res.* In press.

NIFATOV, A. P. & KOSHURNIKOVA, N. A. (1973) Spontaneous Tumours in Wistar Rats. *Vopr. Onkl.*, **19(12)**, 83.

PETO, R. (1974) Guidelines on the Analysis of Tumours Rates and Death Rates in Experimental Animals. *Br. J. Cancer*, **29**, 101.

SCHEVING, L. E., BURNS, E. R. & PAULY, J. E. (1972) Circadian Rhythms in Mitotic Activity and $^{3}$H-thymidine Uptake in the Duodenum: Effect of Isoproterenol on Mitotic Rhythm. *Am. J. Anat.*, **135**, 311.

SCHMUTZ, J. A. & CHAUDHRY, A. P. (1969) Incidence of Induced Tumours in the Rat Submandibular Gland with Different Doses of 7,12-dimethylbenz-(a)anthracene. *J. Dental Res.*, **48**, 1316.

SCOTT, J. F., FRACCASTORO, A. P. & TAFT, E. B. (1956) Studies in Histochemistry: I. Determination of Nucleic Acids in Microgram Amounts of Tissue. *J. Histochem. Cytochem.*, **4**, 1.

SWANN, P. F. (1968) The Rate of Breakdown of Methyl Methane-sulphonate, Dimethylsulphate and *N*-methyl-*N*-nitrosourea in the Rat. *Biochem. J.*, **110**, 49.

WINTER, W. A. (1974) Induction of DNA Synthesis by Isoproterenol in the Rat Urinary Bladder Epithelium. *Histochemistry*, **41**, 141.